# Temporal Dissociation of Impaired Glucose Tolerance, Adipose Lipid Remodeling and Endothelial Dysfunction in Aorta After HFD Withdrawal

**DOI:** 10.1096/fj.202600102RR

**Published:** 2026-04-25

**Authors:** Krzysztof Czamara, Izabela Czyzynska‐Cichon, Anna Bar, Ewa Stanek, Mateusz Wawro, Marta Z. Pacia, Zeinep Berkimbayeva, Brygida Marczyk, Elvira Bragado‐García, Paloma Palma‐Guzman, Maria S. Fernandez‐Alfonso, Stefan Chlopicki

**Affiliations:** ^1^ Jagiellonian Centre for Experimental Therapeutics (JCET) Jagiellonian University in Kraków Kraków Poland; ^2^ Department of Cell Biochemistry, Faculty of Biochemistry, Biophysics and Biotechnology Jagiellonian University in Kraków Kraków Poland; ^3^ Instituto Pluridisciplinar and Faculty of Pharmacy Universidad Complutense de Madrid Madrid Spain; ^4^ Department of Pharmacology Jagiellonian University Medical College in Krakow Kraków Poland

**Keywords:** endothelium, glucose tolerance, guanylate cyclase, high‐fat diet reversal, obesity, perivascular adipose tissue, vascular biology

## Abstract

Glucose intolerance and endothelial dysfunction are metabolic syndrome hallmarks induced by fat load, but their reversibility is not well explored. Here, we characterize the effects of high‐fat diet (HFD) withdrawal on systemic glucose tolerance, endothelial function in aorta (thoracic, TA and abdominal, AA), lipid unsaturation of perivascular adipose tissue (PVAT), and mRNA expression profile. We studied the early and late effects of diet change on C57BL/6J mice after 8 weeks of HFD feeding (60 kcal% of fat with 1% of cholesterol) by implementing multimodal functional (in vivo MRI), spectroscopic (Raman spectroscopy), and molecular (RT‐qPCR) characterization of aortas and PVATs. As soon as 1 week after HFD withdrawal, glucose tolerance was normalized and accompanied by the reversal of lowered *Scd1* gene expression in PVAT and lipid unsaturation index in PVAT of AA, but not of TA. In contrast, impaired acetylcholine‐induced vasodilation in aorta, implicating endothelial dysfunction, was only partially reversed 1 week after HFD withdrawal, whereas full restoration occurred after 6 weeks, with slightly earlier recovery in TA compared to AA. Delayed reversal of endothelial dysfunction in aorta after HFD withdrawal was associated with transcriptomic alterations, indicating downregulation of soluble guanylate cyclase signaling, compensatory changes in insulin signaling, and changes in adipokines expression in PVAT, but not in aorta itself. In summary, HFD withdrawal resulted in early restoration of glucose tolerance, and PVAT lipid unsaturation determined by *Scd1* that was temporarily dissociated from the reversal of endothelial dysfunction in the aorta possibly due to altered PVAT function featured by impaired guanylate cyclase signaling pathway.

## Graphical abstract

HFD withdrawal results in early restoration of systemic glucose tolerance and PVAT lipid unsaturation that is temporarily dissociated from the reversal of endothelial dysfunction in aorta that may be linked to altered PVAT function featured by impaired guanylate cyclase signaling pathway.

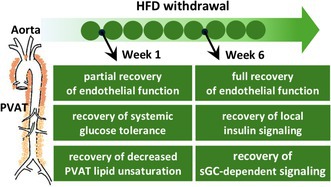

**Abbreviations**
AA: abdominal aorta
Ach: acetylcholine
AUC: area under the curve
BAT: brown adipose tissue
cGMP: cyclic guanosine monophosphate
Et‐1: endothelin 1 encoded by *Edn1*
eWAT: epididymal white adipose tissue
GTC: guanidinium thiocyanate
GTT: glucose tolerance test
HFD: high‐fat diet
Insr: insulin receptor encoded by *Insr*
Irs1/2: insulin receptor substrate 1/2 encoded by *Irs1/2*
MRI: magnetic resonance imaging
Nox2/4: NADPH oxidase 2/4 encoded by *Nox2/4*
Pi3k: phosphatidylinositol 3‐kinase encoded by *Pi3kr1*
Polr2b: RNA polymerase II subunit B encoded by *Polr2b*
Pparg2: peroxisome proliferator‐activated receptor gamma isoform 2 encoded by *Pparg2*
PVAT: perivascular adipose tissue
Scd1: stearoyl‐CoA desaturase 1 encoded by *Scd1*
sGC: soluble guanylate cyclase encoded by *Gucy1b1*
SNP: sodium nitroprusside
Srebp‐1c: sterol regulatory element‐binding protein 1c
TA: thoracic aorta
TG: triacylglycerols
Tnf: tumor necrosis factor encoded by *Tnf*
Ucp1: uncoupling protein 1 encoded by *Ucp1*
WAT: white adipose tissue

## 1 Introduction

The global pandemic of obesity and associated metabolic disorders is driven by a diet rich in saturated fat, a high‐fat diet (HFD) or so‐called Western diet, and a sedentary lifestyle. HFD affects glucose homeostasis and insulin sensitivity, leading to systemic insulin resistance [1], impairs endothelial function [2] and causes perivascular adipose tissue (PVAT) dysfunction [3]. The last decades have revealed that PVAT, surrounding the aorta, considerably contributes to cardiovascular, lifestyle, and age‐related diseases [4].

PVAT is anatomically and functionally distinct due to its direct proximity to blood vessels and its local paracrine effects on vascular tone and inflammation whereas a classical visceral white adipose depot, that is, epididymal white adipose tissue (eWAT), is primarily involved in energy storage and endocrine regulation [5]. In healthy individuals, PVAT has a protective, anti‐contractile, and anti‐inflammatory profile, and secretes various vasoactive molecules, including adipocyte‐derived NO, which signal through guanylate cyclase (GC) pathway, and adipokines, that is, leptin and adiponectin, contributing to the regulation of vascular signaling and endothelial function [6]. Soluble guanylate cyclase (sGC), the canonical NO receptor, is a central mediator in the cyclic guanosine monophosphate (cGMP)‐dependent vasorelaxation cascade [7]. However, when PVAT becomes dysfunctional, it increases reactive oxygen species level, releases pro‐inflammatory cytokines and vasoconstrictor factors, leading to oxidative stress, macrophage infiltration, redox imbalance, thus, promotes endothelial dysfunction [8, 9] and insulin resistance [10]. Moreover, pro‐inflammatory and pro‐oxidative PVAT decreases NO bioavailability, causing impairment in NO‐sGC‐cGMP signaling, leading to disrupted PVAT–vascular crosstalk [11]. The negative effects of HFD, including depot‐specific PVAT dysfunction, are characterized by increased inflammation, disrupted metabolic flexibility, excessive lipid accumulation, adipocyte hypertrophy, and loss of vascular protection, all of which promote the development of cardiovascular disease [12].

Current first‐line strategy to reverse metabolic dysfunction includes, inter alia, non‐invasive lifestyle interventions, increased physical activity, and pharmacological treatment or invasive bariatric surgery [13, 14, 15]. Some positive reversal effects, that is, body weight reduction, improved glucose homeostasis, and restoration of insulin sensitivity, can be achieved only by HFD withdrawal and return to a normal diet [16, 17]. However, it is still unclear how rapidly insulin sensitivity, PVAT lipid profile, and endothelial function are normalized following HFD withdrawal, and what role PVAT remodeling plays in the reversal to vascular homeostasis. Thus, in this work, we aimed to define time‐dependent reversal of HFD‐induced impairment of glucose tolerance and PVAT biochemical changes in relation to vascular endothelial (dys)function after HFD withdrawal and reveal the molecular mechanism responsible for observed alterations by implementing multimodal functional, spectroscopic, and molecular characterization.

Due to PVAT's specific location and heterogeneity, there is a limited number of established techniques for its investigation [18]. Apart from several approaches based on image acquisition, that is, PET/CT or MRI techniques [19], PVAT chemical characteristics can be provided by Raman spectroscopy, a label‐free and unbiased technique, which enables the analysis of the chemical composition, especially in the case of lipids that have a large cross‐section for Raman scattering [20]. Relevant Raman markers, such as the lipid unsaturation degree, defined as the ratio of integral intensities of bands at ca. 1660 to 1444 cm^−1^, were established to characterize qualitatively and quantitatively lipids in PVAT [21, 22]. MRI‐based methodology enabled in vivo assessment of aortic endothelium‐dependent acetylcholine (Ach)‐induced vasodilation in the presence of PVAT, in contrast to wire myography when PVAT is removed [23]. In our previous work we showed that after a short, two‐week HFD period, endothelial dysfunction assessed by MRI was present only in the abdominal aorta (AA), while the thoracic aorta (TA) remained unchanged due to the brown‐like phenotype of TA PVAT, highlighting the protective role of adjacent PVAT [23].

Here, taking advantage of these unique methodologies, we characterized the early recovery of PVAT lipid unsaturation degree by Raman spectroscopy and in vivo aortic endothelium function by MRI after 8 weeks of HFD feeding and subsequent 6 weeks of HFD withdrawal. We described the dissociation between early reversal of impaired glucose tolerance and delayed PVAT phenotype‐linked reversal of endothelial dysfunction in the aorta after HFD withdrawal, pointing to possible mechanisms underlying systemic and local regulation of PVAT phenotype.

## 2 Materials and Methods

### 2.1 Animals

Studies were performed in 12‐week‐old C57BL/6J male mice (Mossakowski Medical Research Centre, Polish Academy of Sciences, Warsaw, Poland), fed an HFD (60 kcal% of fat + 1% of cholesterol; ZooLab, Krakow, Poland) for 8 weeks and an AIN‐93G diet (normal diet, ZooLab, Poland) as a control. Then, HFD was changed to an AIN‐93G diet for 1, 2, 4, and 6 weeks. Animals had ad libitum access to daily‐provided diets and water. The experiment involved 8 animals per group, and mice were randomly distributed into groups. The attrition rate in our study was 2% (*n* = 1 of the control group). Mice were housed in collective cages, in a room with optimal conditions (cage temperatures of 22°C ± 2°C, 55% ± 10% humidity, 12‐h light/dark cycle, and 75 air changes per hour filtered by a set of filters). Animals were weighed at the start and regularly checked each week until the end of the experiment. Data were presented as body weight and body weight percentage changes after the respective diet duration. All experiments were approved by the local ethics committee of Jagiellonian University (Krakow, Poland, identification code: 246/2023) and were in accordance with the Guide for the Care and Use of Laboratory Animals of the National Academy of Sciences (National Institutes of Health publication No. 85–23, revised 1996), as well as the Guidelines for Animal Care and Treatment of the European Community.

### 2.2 Diet Composition

High fat diet (HFD, 60 kcal% of fat +1% of cholesterol, ZooLab, Krakow, Poland) was selected according to our previous study [23]. HFD was formulated based on standard AIN‐93G, and both diets' compositions and nutrition facts were displayed in Table S1, ESI.

### 2.3 Inclusion/Exclusion Criteria

The animals in HFD withdrawal groups were included in the study if they, after 8 weeks of HFD feeding, significantly increased body weight compared to the control group.

### 2.4 Assessment of Insulin Resistance Based on Glucose Tolerance Test (GTT)

Before GTT was performed, mice were fasted for 4 h with only access to water. Basal glucose level was measured from a blood drop from the tail, cut at the end using a standard glucometer. Blood glucose concentration measurements were repeated after 15, 30, 60, and 120 min of intraperitoneal glucose administration (2 g/kg body weight). Results are presented as a curve of blood glucose concentration over time and as the calculated area under the curve (AUC).

### 2.5 Assessment of Acetylcholine‐Induced Vasodilation In Vivo by MRI

MRI experiments were performed using a 9.4 T scanner (BioSpec 94/20, USR). During measurements, mice were anesthetized using isoflurane (Aerrane, Baxter Sp. z o. o., 1.5 vol%) in oxygen and air (1:2) mixture and imaged in the supine position. Heart function (rhythm and ECG), respiration and body temperature (maintained at 37°C using circulating warm water) were monitored using a Monitoring and Gating System (SA Instruments Inc.). Endothelial function in vivo was assessed by a previously described technique [23] as an endothelium‐dependent response to acetylcholine (Ach, Sigma‐Aldrich; 50 μL, 16.6 mg/kg, IP), while the vascular smooth muscle cell–dependent response was measured following administration of sodium nitroprusside (SNP, Sigma‐Aldrich; 1 mg/kg, IV). Both responses were analyzed in the AA and TA by comparing two, time‐resolved 3D images of the vessels before and 30 min after compounds administration. The dose of Ach and optimal time to measure Ach‐induced vasorelaxation were chosen based on our previous work [23]. 3D images of the aorta were acquired using the cine IntraGate FLASH 3D sequence and reconstructed with the IntraGate 1.2.b.2 macro (Bruker). Analysis was performed using ImageJ software 1.46r (National Institutes of Health) and scripts written in MATLAB (MathWorks), in the hyperstack of the AA (10 slices in diastole, from the renal arteries down) and the TA (10 slices in diastole, from the celiac artery up). All cross‐sectional areas of vessels at each slice were obtained using thresholding segmentation and exported to MATLAB, where vessel volumes were reconstructed and calculated. Imaging parameters included the following: 6.4 ms repetition time, 1.4 ms echo time, 30 × 30 × 14 mm [3] field of view, matrix 256 × 256 × 35 size, 30° flip angle, and 15 number of accumulations, reconstructed to 7 cardiac frames. Total scan time was about 12 min.

### 2.6 Tissue Collection and Blood Biochemistry

Animals were anesthetized by intraperitoneal injection of a mixture of ketamine and xylazine at 100 mg ketamine/10 mg xylazine/kg body weight. After sacrifice, blood was collected directly from the heart into the syringe containing 10% solution of EDTA dipotassium salt (Aqua‐Med; 1 μL of EDTA/100 μL of collected blood). All samples were centrifuged at 664 *g*, at 4°C for 10 min to isolate plasma, then stored at −80°C until lipid profile measurement. Lipid profiles (TG, TCHOL, HDL, and LDL) were analyzed in plasma samples using an ABX Pentra 400 automated biochemistry analyzer (Horiba, Kyoto, Japan) according to the manufacturer's instructions. Successively, the liver and epididymal white adipose tissue (eWAT) were extracted and weighed. In the last stage, the entire aorta was extracted and transferred into NaCl injection solution. Subsequently, aortas were divided into TA and AA based on diaphragm location and cleaned of surrounding PVAT. Such prepared aortas and PVAT samples were preserved in nucleic acid preservation buffer (NAP), prepared according to ref. [24] and stored at −80°C for later RNA isolation and RT‐qPCR analysis. Additionally, small fragments of TA and AA PVAT were kept for Raman spectroscopy.

### 2.7 Characteristics of PVAT of the TA and AA by Raman Spectroscopy

Raman spectra of fresh unfixed TA and AA PVAT samples placed on CaF_2_ slides were measured on the day of isolation using the Raman spectrometer WITec Alpha300 (Ulm, Germany) equipped with an air‐cooled solid‐state laser with an excitation wavelength of 532 nm, a UHTS 300 spectrograph (600 grooves per mm grating), a CCD detector (DU401A‐BV‐352, Andor, UK), and a 20× air objective (NA = 0.45, Nikon, Japan). To study the HFD recovery changes in PVAT samples, at least seven good‐quality spectra for each sample were taken for analysis. For each Raman spectrum, 10 accumulations were acquired with a 0.5 s exposure time per spectrum using the maximum laser power at the sample (ca. 30 mW).

Data preprocessing was done using the WITec Project Plus software (WITec, Ulm, Germany) and included an autopolynomial of degree 3 baseline‐correction procedure. Then, Raman spectra were normalized using vector normalization in the 1800–400 cm^−1^ spectral region using the OPUS 7.2 software (Billerica, MA, USA). The integral intensities of the bands 1657 and 1441 cm^−1^ for Raman spectra of PVAT samples were calculated and used to determine the degree of lipid unsaturation (I_1657_/I_1441_).

### 2.8 RNA Isolation and Reverse Transcription

TA, AA, and corresponding PVATs tissue samples were taken out of the NAP buffer and dried on tissue paper, then homogenized in a 1:1 phenol (pH = 4.0): GTC mixture (4 M guanidinium thiocyanate, 25 nM sodium citrate, pH 7.0, 0.05% (w/v) sarkosyl, and 1% (v/v) 2‐mercaptoethanol). Total RNA isolation was performed according to Chomczynski‐Sacchi's modified protocol [25]. To increase the yield of total RNA, samples were coprecipitated with linear acrylamide (Thermo Fisher Scientific) at a final working concentration of 10 μg/mL in 3 M sodium acetate (pH 4.3). RNA quantification and purity were assessed using spectrophotometric measurements using a NanoDrop ND‐1000 spectrophotometer (Thermo Fisher Scientific). RNA integrity was assessed by denaturing, formaldehyde gel electrophoresis. Reverse‐transcription reaction was performed using 500 ng of RNA with M‐MLV‐Reverse transcriptase (Promega) and 250 ng of a 1:1 mixture of oligo(dT) primers (Genomed) and random hexamers (EurX) according to the manufacturer's instructions. The cDNA was used in quantitative PCR (RT–qPCR) for the evaluation of the amounts of the mRNAs of interest.

### 2.9 RT‐qPCR Analysis of PVAT and Aortas

The RT‐qPCR was performed using qPCR‐HS Mix SYBR (A&A Biotechnology). Levels of the mRNAs of interest in each sample were analyzed using the Pfaffl method and the expression level was normalized to the *Polr2b* expression level. Primer specificity was tested in 2% agarose gel electrophoresis and efficiencies were calculated using the serial dilution method. The sequences of primers were collected in Table S2, ESI. Statistical analysis of RT‐qPCR obtained results (one‐way ANOVA with Tukey's multiple comparisons test) was performed on the logarithm‐transformed relative quantification values [26]. Pair comparison (*t*‐test) between TA and AA depots for the same diets, and similarly for PVAT was summarized in Table S3, ESI.

### 2.10 Statistical Analysis

The obtained data are presented as the mean ± standard deviation, with each point representing a single animal. Unless otherwise stated, statistical analyses were performed in the GraphPad Prism 10 program using the one‐way ANOVA with the Tukey post hoc test. If the P‐value was at most 0.05, differences were identified as statistically significant. The correlation analysis was performed for selected parameters from mice in all diet groups using a two‐tailed nonparametric Spearman correlation.

## 3 Results

### 3.1 Dissociation Between Impaired Glucose Tolerance and Endothelial Function in Aorta After HFD Withdrawal in Mice

**FIGURE 1:**
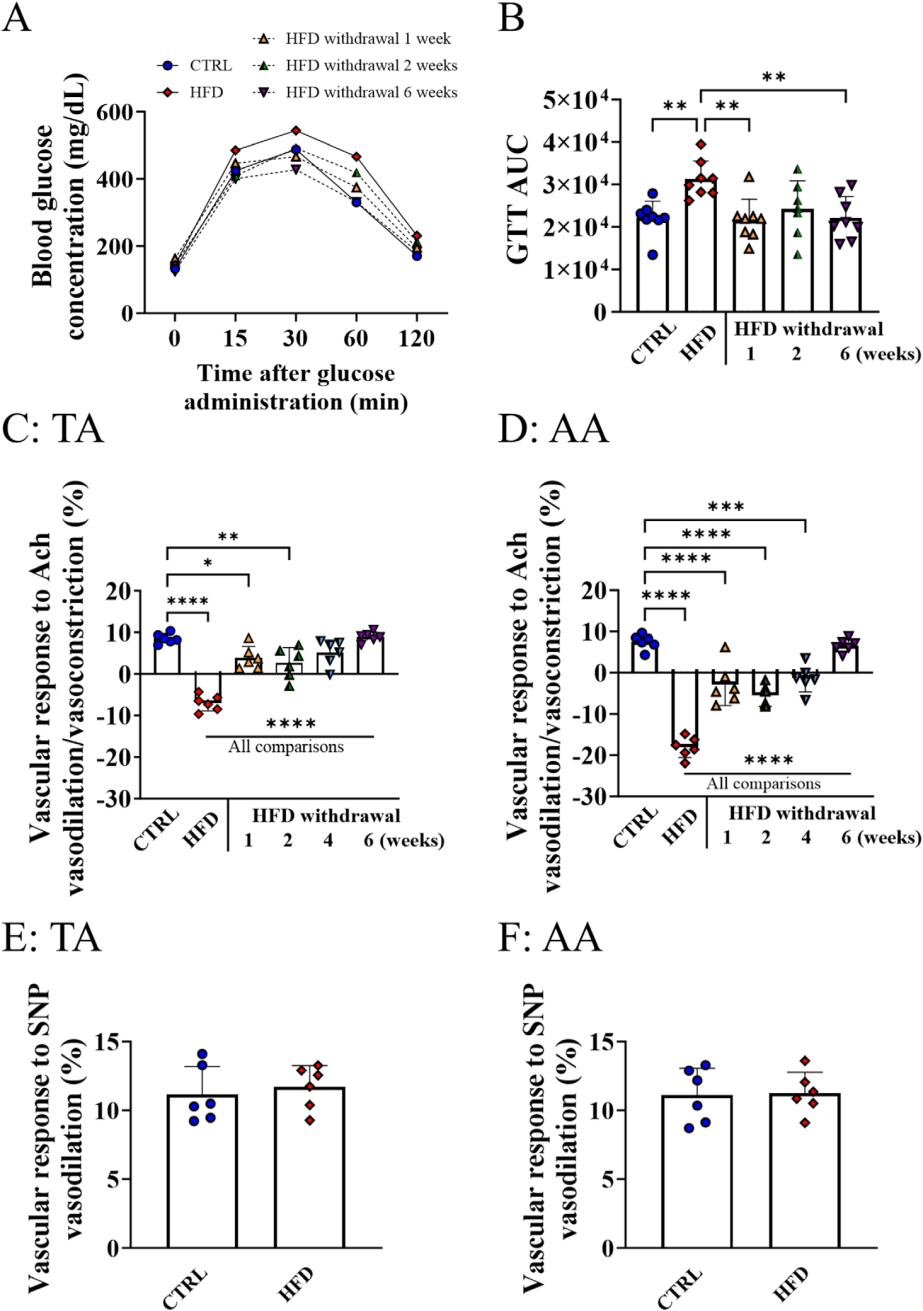
Effects of 8 weeks of HFD and time‐dependent effects of HFD withdrawal on glucose tolerance and endothelial function in aorta. Changes in blood glucose concentrations after 2 g/kg glucose injection in glucose tolerance test (A, GTT) and area under the GTT curve (B) in C57BL/6J mice after HFD reversal to normal diet for 1, 2, and 6 weeks. Changes in the vessel volume in response to acetylcholine (Ach) measured for the TA (C) and AA (D) in C57BL/6J mice fed an HFD for 8 weeks and after replacement of HFD by AIN‐93G diet for 1 2, 4 and 6 weeks, and response to sodium nitroprusside (SNP) measured for the TA (E) and AA (F) for C57BL/6J mice fed an HFD for 8 weeks in comparison to C57BL/6J mice fed a standard AIN‐93G diet. Data are represented as mean ± SD of 6–8 determinations per group. Statistics: One‐way ANOVA with Tukey post hoc test, *p < 0.05, **p < 0.01, ***p < 0.001. ****p < 0.0001.

HFD feeding for 8 weeks resulted in obesity manifested by elevated body weight, increased by 15% (Figure S1). 8 weeks of HFD also induced alterations in lipid profile (Figure S1) and glucose tolerance as evidenced by increased area under the GTT curve (~1.5 fold higher for HFD group compared to control group) with no changes in basal glucose plasma level in fasting conditions (Figure 1A). HFD withdrawal reversed impaired glucose tolerance to normal as soon as after 1 week, an effect maintained up to 6 weeks (Figure 1A,B).

HFD given to C57BL/6J mice for 8 weeks resulted in endothelial dysfunction as evidenced by Ach‐induced vasoconstriction in TA and AA (Figure 1C,D), with a significantly more pronounced effect in AA (volume change decreased to −18% compared to −7% for TA). In contrast to the rapid reversal of glucose homeostasis after HFD withdrawal, HFD‐induced endothelial dysfunction was not fully reversed after 1 week (volume changes increased to 3.8% in TA and −2.9% in AA), and required 6 weeks to be progressively restored to normal values (volume change of 8% in TA and 7% in AA). Importantly, the endothelium‐independent response induced by SNP in the AA and TA was unchanged after 8 weeks of HFD in C57BL/6J mice (Figure 1E,F).

### 3.2 Negative Correlation Between Glucose Tolerance and PVAT Lipid Composition After HFD Withdrawal in Mice; Role of *Scd1*

**FIGURE 2:**
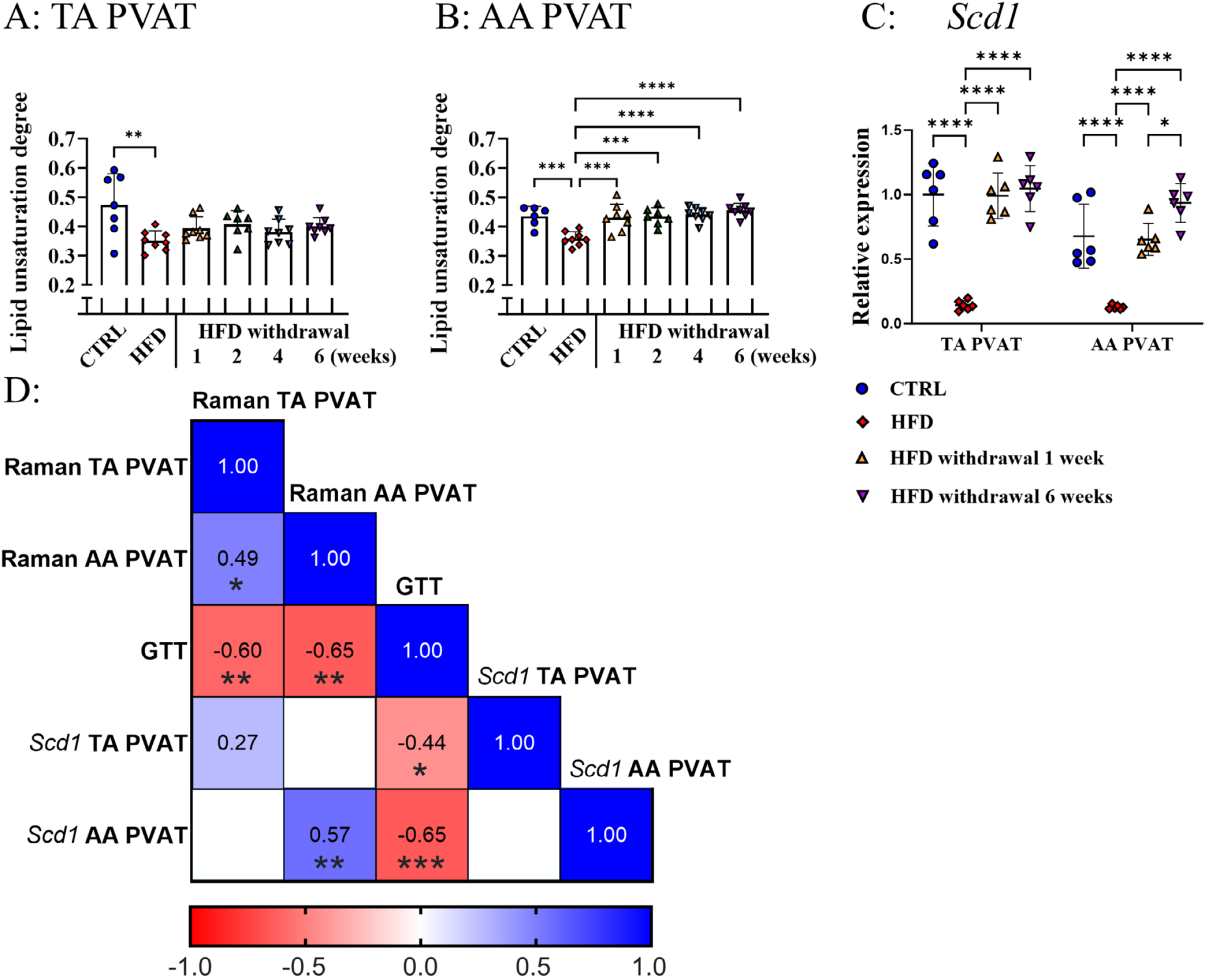
Effects of 8 weeks of HFD and time‐dependent effects of HFD withdrawal on lipid unsaturation degree and mRNA expression of genes related to lipid handling in PVAT. Raman spectroscopy analysis of lipid unsaturation degree (I_1657_/I_1441_) changes of TA and AA PVAT (A and B, respectively) and mRNA level of *Scd1* (C) evoked by diet reversal after 8 weeks of HFD to C57BL/6J mice. The correlation matrix of Raman marker, *Scd1* gene expression in PVAT and GTT (D). Data are represented as mean ± SD of 6–8 determinations per group. Statistics: (A, B) One‐way ANOVA with Tukey post hoc test, (C) One‐way ANOVA with Tukey post hoc test, data were log_10_‐transformed for statistical analysis; raw values are shown. *p < 0.05, **p < 0.01, ***p < 0.001, ****p < 0.0001.

HFD feeding for 8 weeks modified the lipid composition of PVAT and lipid unsaturation degree as revealed by Raman spectroscopy (Figure 2, Figure S2). HFD withdrawal caused normalization of lipid unsaturation degree to the control group's level in AA PVAT already after 1 week, while in TA PVAT, only a slight increase (by ca. 15%) was observed (Figure 2A,B). Interestingly, 8 weeks of HFD downregulated gene expression of *Scd1*, a rate‐limiting enzyme in the formation of monounsaturated fatty acids, in both TA and AA PVAT, and its expression was fully normalized as soon as 1 week of HFD withdrawal (Figure 2C). Additionally, Spearman's rank correlation analysis revealed that lipid unsaturation degree has a negative correlation with the results of GTT (*R* > −0.60, *p* < 0.01) and lipid unsaturation degree for AA PVAT showed a positive correlation (*R* = 0.57, *p* < 0.01) with *Scd1* gene expression (Figure 2D).

### 3.3 mRNA Expression Profile of Thoracic and Abdominal Aorta After HFD Withdrawal in Mice

**FIGURE 3:**
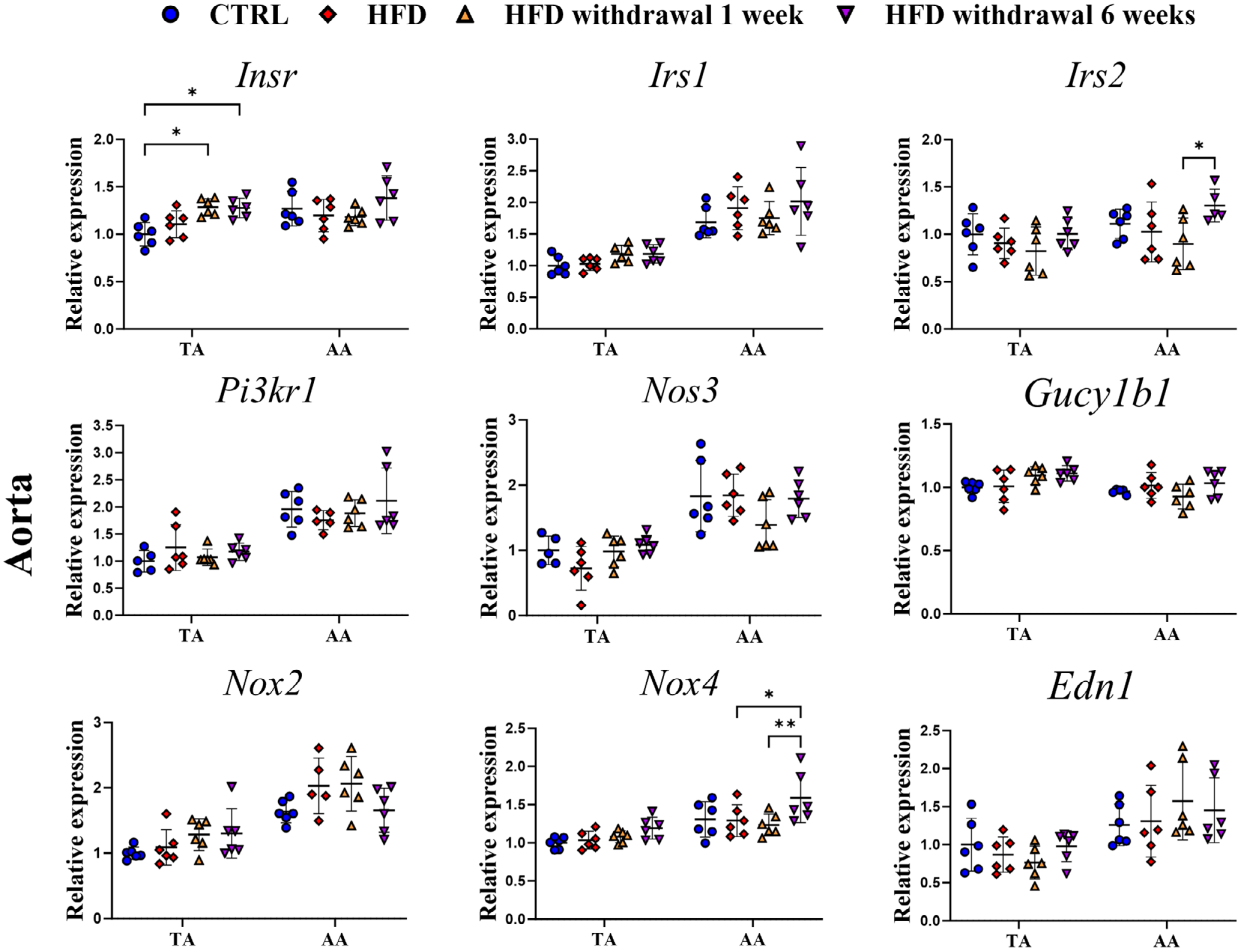
Effects of 8 weeks of HFD and time‐dependent effects of HFD withdrawal on mRNA expressions of genes related to the insulin pathway in aorta. mRNA levels of *Insr*, *Irs1/2*, *Pi3kr1, Nos3, Gucy1b1, Nox2, Nox4*, and *Edn1* in TA and AA from C57BL/6J mice fed an HFD for 8 weeks and after replacement of HFD by AIN‐93G diet for 1 and 6 weeks in comparison to C57BL/6J mice fed a standard AIN‐93G diet for 8 weeks. Data are represented as line plots: Mean ± SD of 5–6 determinations per group. Statistics: One‐way ANOVA with Tukey post hoc test; data were log_10_‐transformed for statistical analysis; raw values are shown, *p < 0.05, **p < 0.01.

To verify whether delayed reversal of endothelial function after HFD withdrawal was associated with the local changes in the mRNA expression profile of the aorta, several genes related to insulin signaling were analyzed in the aorta (Figure 3).

*Nos3, Gucy1b1*, and *Edn1*, genes involved in vascular function and endothelial signaling, contributing to the regulation of blood pressure and vascular tone, were not altered after HFD and 1 and 6 weeks after HFD withdrawal. Moreover, *Nox2* and *Nox4* expression in TA was not changed either. Among genes related to insulin signaling (*Insr*, *Irs1/2*, *Pi3kr1*), only the expression of *Insr* in the TA was upregulated after 1 and 6 weeks of HFD withdrawal. In contrast, the AA did not exhibit upregulation of genes related to insulin signaling until 6 weeks after HFD reversal, when *Irs2* was upregulated, accompanied by an increase in *Nox4* expression.

### 3.4 mRNA Expression Profile of Thoracic and Abdominal PVAT After HFD Withdrawal in Mice

**FIGURE 4:**
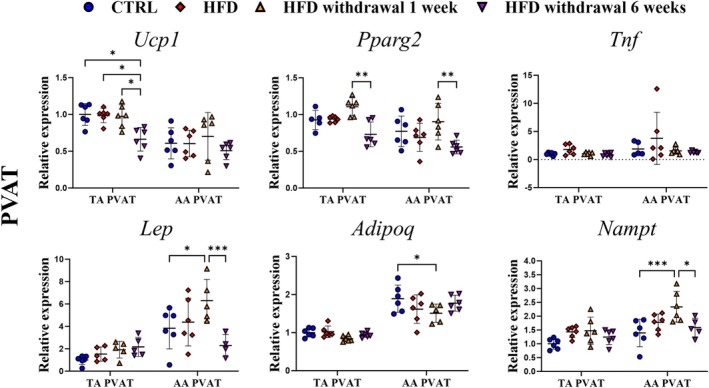
Effects of 8 weeks of HFD and time‐dependent effects of HFD withdrawal on mRNA expressions of adipokines and lipid metabolism‐related genes in PVAT. mRNA levels of *Ucp1, Pparg2, Tnf, Lep*, *Adipoq*, and *Nampt* in TA and AA PVAT from C57BL/6J mice fed an HFD for 8 weeks and after replacement of HFD by AIN‐93G diet for 1 and 6 weeks in comparison to C57BL/6J mice fed a standard AIN‐93G diet for 8 weeks. Data are represented as line plots: Mean ± SD of 5–6 determinations per group. Statistics: One‐way ANOVA with Tukey post hoc test, data were log_10_‐transformed for statistical analysis; raw values are shown. *p < 0.05, **p < 0.01, ***p < 0.001.

Given that no substantial transcriptomic changes were observed in the aorta induced by HFD and HFD withdrawal, possible alterations in genes related to adipose tissue phenotype and function in PVAT (Figure 4) were explored.

As shown in Figure 4, transcriptomic analysis revealed the heterogeneity of PVAT phenotype, defined by higher gene expression of *Ucp1* and *Pparg2* (BAT markers) in TA PVAT and *Lep* (WAT marker) in AA PVAT. Interestingly, 8 weeks of HFD did not trigger PVAT inflammation assessed by *Tnf*; only a slight increase was observed in AA PVAT and was normalized 1 week after HFD withdrawal. HFD withdrawal resulted in slightly upregulated *Pparg2* expression after 1 week and, together with *Ucp1*, significantly decreased below the control group level after 6 weeks of HFD withdrawal. Adipokine gene expressions were also influenced by HFD withdrawal but only in AA PVAT. *Lep* and *Nampt* slightly increased after HFD and significantly after 1 week of diet withdrawal, followed by downregulation of *Adipoq*. After 6 weeks of HFD withdrawal, all adipokine gene expressions returned to the control level.

**FIGURE 5:**
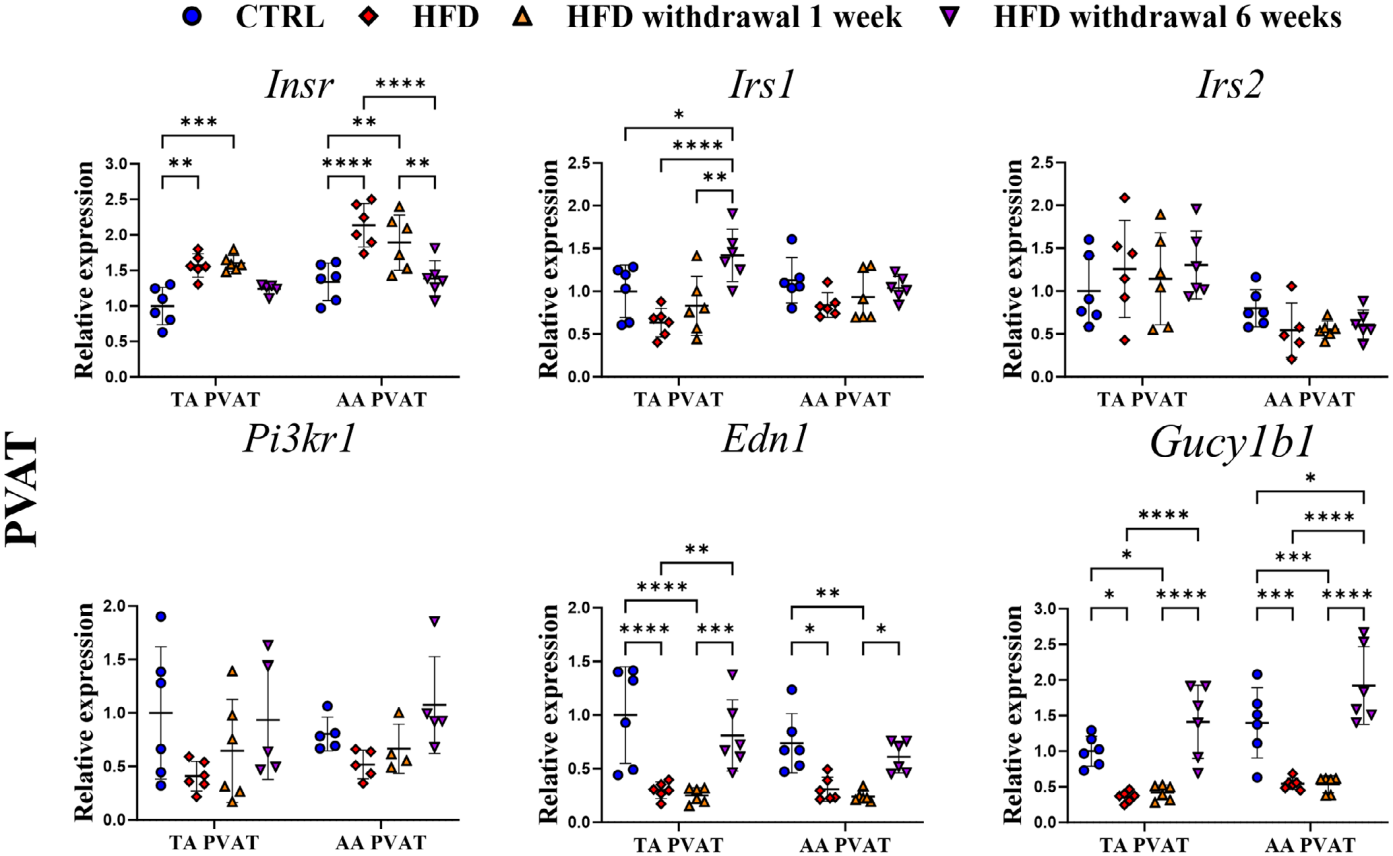
Effects of 8 weeks of HFD and time‐dependent effects of HFD withdrawal on mRNA expressions of genes related to the insulin pathway in PVAT. mRNA levels of *Insr*, *Irs1/2*, *Pi3kr1, Edn1*, and *Gucy1b1* in TA and AA PVAT from C57BL/6J mice fed an HFD for 8 weeks and after replacement of HFD by AIN‐93G diet for 1 and 6 weeks in comparison to C57BL/6J mice fed a standard AIN‐93G diet for 8 weeks. Data are represented as line plots: Mean ± SD of 5–6 determinations per group. Statistics: One‐way ANOVA with Tukey post hoc test, data were log_10_‐transformed for statistical analysis; raw values are shown. *p < 0.05, **p < 0.01.

Expression of genes related to insulin signaling was altered in PVAT by HFD and HFD withdrawal (Figure 5). We observed upregulation of *Insr*, which returned to the control level after 6 weeks of HFD withdrawal. This effect was more pronounced in AA PVAT compared with TA PVAT. Also, in AA PVAT, *Irs1/2* and *Pi3kr1* downregulation after 8 weeks of HFD returned to control level 6 weeks post‐withdrawal, whereas in TA PVAT, *Irs1* expression gradually increased over 6 weeks of diet reversal, with *Irs2* expression unchanged. Interestingly, as shown in Figure 5, HFD induced a substantial downregulation of *Edn1* and *Gucy1b1* in PVAT, and the effects were similar for AA and TA PVAT. HFD withdrawal did not reverse these changes after 1 week, but the full reversal of downregulated *Edn1* and *Gucy1b1* expression to the control level was observed after 6 weeks, temporally coinciding with the reversal of endothelial dysfunction after HFD withdrawal.

## 4 Discussion

In the present work, we demonstrated that HFD withdrawal resulted in early reversal of disturbed glucose homeostasis and normalization of PVAT lipid unsaturation, correlating with *Scd1* expression. However, reversal of endothelial dysfunction in aorta was delayed and transcriptomic analysis, specifically in PVAT but not in the aortic vascular wall, suggested local insulin resistance and parallel impairment of NO‐sGC‐cGMP signaling. HFD withdrawal revealed a temporal dissociation between glucose tolerance, PVAT lipid remodeling, and vascular endothelium function recovery. These results highlight guanylate cyclase in PVAT as a possible key target for reversing HFD‐induced endothelial dysfunction and restoring vascular homeostasis.

Here, the early effects of HFD withdrawal were observed as soon as 1 week after diet exchange, as evidenced by a full reversal of impaired glucose tolerance together with normalized plasma TG and HDL, highlighting quick systemic recovery of glucose and lipid metabolism after HFD withdrawal. Moreover, at the same time, HFD withdrawal resulted in the full recovery of changes in lipid unsaturation degree in PVAT of AA; however, that was not observed in PVAT of TA as assessed by Raman spectroscopy. The latter result seems to be compatible with PVAT regional heterogeneity in the aorta arising from the different origin of forming adipocytes [27] resulting in diverse, region‐dependent phenotypes of PVAT in TA and AA. TA PVAT resembles brown adipose tissue (BAT), as indicated by its high expression of *Ucp1* and *Pparg2*, and is mostly involved to non‐shivering thermogenesis. In contrast, PVAT surrounding the AA exhibits a more heterogeneous phenotype. With higher *Lep* and *Adipoq* expression levels, it exhibits mixed characteristics of both BAT and white adipose tissue (WAT), the latter primarily involved in lipid storage [5]. Our previous research on PVAT using Raman spectroscopy in various disease models [21, 28], enabled us to establish a relevant marker for assessing PVAT lipid composition, the lipid unsaturation degree; however, the biological background of this index has not been determined. Here, we observed decreased lipid unsaturation degree in the entire PVAT after 8 weeks of HFD, contrary to the short‐term HFD feeding model, where only WAT‐like AA PVAT was altered, while TA PVAT of BAT‐like characteristic remained unchanged [21]. Moreover, after 1 week of HFD withdrawal, lipid unsaturation degree was normalized only in the PVAT of AA. Interestingly, it was correlated with insulin resistance status, but did not reflect the vascular endothelium (dys)function status. These results suggested that the lipid profile of PVAT was associated with glucose and fatty acids metabolism, reflecting global changes in metabolism regulated by insulin. Additionally, the correlation analysis revealed a significant positive correlation with *Scd1* in WAT‐type AA PVAT. As the most abundant desaturase in mice adipose tissue, Scd1 acts in maintaining lipid balance, having influence on insulin signaling and sensitivity, protecting cells from saturated fatty acid‐induced toxicity, thereby determining the degree of lipid unsaturation [29]. Scd1 is positively regulated by insulin via activation of Srebp‐1c, which induces its transcription [30], and therefore its downregulation in HFD may result from suppression of de novo lipogenesis due to increased overload and re‐esterification of dietary fatty acids.

In contrast to early recovery of glucose tolerance and PVAT lipid remodeling, reversal of endothelial dysfunction in aorta required 6 weeks after HFD withdrawal, highlighting the independence of systemic glucose homeostasis from HFD‐induced endothelial dysfunction. As evidenced using MRI in vivo measurements, after 1 week of HFD withdrawal, endothelial function was partially reversed with a more pronounced effect in TA, but was not fully recovered. A similar pattern of heterogeneous response of TA and AA was assessed in a humanized dyslipidemia model of E3L.CETP male mice, indicating the protective role of PVAT in TA [31]. Importantly, profile of endothelial response was dissociated from a quick reversal of disturbed glucose homeostasis, revealing that endothelial function recovery was not associated with systemic insulin resistance, which redirected our attention to local triggers. Our data showed that alterations in PVAT function may be associated with impaired sGC signaling pathway. Indeed, a significant decrease of *Gucy1b1* expression encoding β1 of sGC after HFD and normalization not sooner than 6 weeks after HFD withdrawal was observed. Although the role of sGC in PVAT has not been extensively studied, there is evidence that this enzyme plays a pivotal role in regulating adipose tissue function. In WAT, the obesity‐induced suppression of sGCβ1 reduced cGMP cascades, impaired adipocyte differentiation, disrupted PPARγ‐driven gene programs, and was associated with inflammation [32]. Pharmacological activation of sGC with praliciguat triggered anti‐inflammatory effects in eWAT [33] while treatment with liraglutide induced WAT browning via upregulation of sGC and protein kinase G I (PKG I), boosting thermogenic gene expression and mitochondrial biogenesis [34]. In BAT, genetic deletion of the heme‐containing β1 subunit of sGC impaired thermogenic programming, whereas pharmacological activation of sGC activated BAT [35] and increased *Edn1* expression in eWAT of obese mice [33]. Moreover, the NO‐sGC‐cGMP pathway was not restricted to thermogenesis, but also involved the regulation of lipolytic responses in 3T3‐L1 adipocytes under endothelin‐1 (Et‐1) or TNF influence [36] and induced perivascular progenitor cells differentiation into brown adipocytes [37]. Conversely, in the metabolic syndrome/HFpEF model, oxidative stress and miRNA‐mediated downregulation of sGCβ1 compromised NO–cGMP signaling and exacerbated endothelial and vascular dysfunction [38]. Together, these findings underscore the importance of NO‐sGC‐cGMP signaling in the regulation of adipose tissue. In this context, our study is the first to suggest that sGC may act as an integral component, possibly linking PVAT phenotype and endothelial function upon HFD withdrawal.

Although the mechanisms of the delayed recovery of sGC in HFD withdrawal and its mechanistic link to endothelial dysfunction recovery were not determined here, we provide evidence that it was not linked to vascular insulin resistance [39]. Our results suggested even possibly more effective insulin vascular signaling, through the Insr‐Irs1/2‐Pi3k pathway in TA, as evidenced by the upregulated expression of *Insr* in TA already at week 1 after HFD withdrawal. Upregulation of *Irs2* in AA was not observed until 6 weeks after HFD withdrawal. Additionally, the temporal changes in *Nox4* expression and no differences for *Nox2* expression between groups suggest that delayed recovery of endothelial function after HFD withdrawal was not directly linked to prolonged increased NO inactivation by superoxide anion. Additionally, in vascular insulin‐resistant states, vascular tone can be modulated by Et‐1, a potent vasoconstrictor that favors vasoconstriction through an enhancement of MAPK signaling and interfering with the Insr‐Irs1/2‐Pi3k‐eNOS‐sGC pathway, thus contributing to endothelial dysfunction [40]. However, we can exclude a deleterious role for *Edn1*, since expression levels were similar in both TA and AA. Interestingly, *Edn1* expression in both PVATs was significantly decreased in HFD and was normalized after 6 weeks of HFD reversal. Et‐1 exerts a dual action that contributes to the fine‐tuning of vascular tone and endothelial function [41]. It induces vasoconstrictive effects primarily through Et_A_ receptors on vascular smooth muscle, while activation of endothelial Et_B_ receptors promotes vasodilation via NO and prostacyclin release. Our findings contrast with a recent study [42] that shows Et‐1 as a major contributor to PVAT dysfunction in obesity and that pharmacological Et_A_ blockade restores PVAT's cardiovascular protective role. The altered expression of vascular‐related genes in PVAT may predominantly originate from endothelial cells or pericytes, suggesting microvascular dysfunction within the adipose tissue niche or be attributed to differences in diet composition and/or duration of HFD experiment. However, further analyses would be required to confirm cell‐type specific effects of Et‐1.

Delayed recovery of endothelial function after HFD withdrawal displayed a slight temporal difference between TA and AA, compatible with heterogeneity of periaortic PVAT phenotype, defined by different gene expressions of BAT and WAT markers [43]. In our study, *Ucp1* and *Pparg2* transcript levels, marker genes for brown‐like TA PVAT, revealed that no alterations were evoked by HFD, but significant downregulation of both was observed as late side effects upon HFD reversal. Interestingly, after 6 weeks of HFD withdrawal *Ucp1* in TA PVAT decreased below the control group level, indicating a loss in brown type phenotype, even when all other parameters were reversed. For other genes, such as *Scd1*, *Lep* and *Insr*, which showed differential expression between TA and AA PVAT under control conditions, HFD followed by its withdrawal led to a more uniform expression pattern, effectively diminishing the regional heterogeneity. These results pointed out that HFD can cause long‐lasting transcriptomic and epigenetic modifications regardless of noticeable weight reduction and improved insulin sensitivity [44]. In contrast, in AA PVAT disturbed adipokine profile revealed increased expression of *Lep* (leptin) and *Nampt* (visfatin), together with decreased *Adipoq* (adiponectin), suggesting a pro‐inflammatory and insulin‐resistant environment [45], and were restored only after 6 weeks of HFD reversal. Leptin resistance may further impair metabolic and vascular responses, and visfatin has been associated with endothelial dysfunction and vascular inflammation [46], while the decrease in adiponectin, an anti‐inflammatory and vasoprotective adipokine, impacts proper vascular endothelial function and impairs glucose uptake [47]. Interestingly, alterations of adipokine gene expression did not intensify endothelial dysfunction in the context of dietary normalization but may be responsible for the delay in the recovery of endothelial function between TA and AA. Heterogeneity of TA and AA PVAT was also seen at different gene levels of insulin downstream signaling in response to HFD reversal, indicating better recovery status for TA and the possible development of local insulin resistance in AA PVAT, which may lead to microvascular dysfunction and capillary rarefaction [48, 49, 50].

**FIGURE 6:**
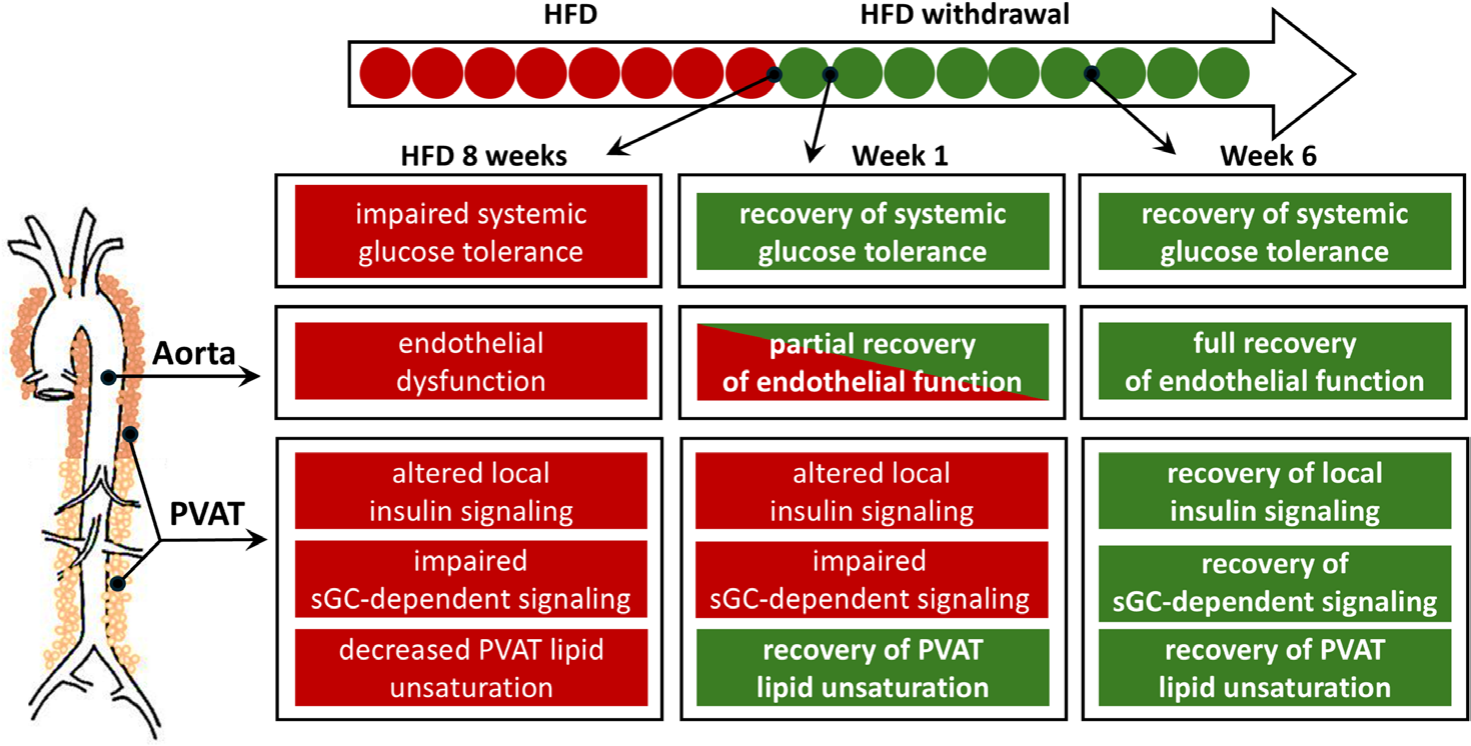
Schematic diagram summarizing evidence for temporal dissociation between recovery of glucose tolerance, perivascular adipose lipid remodeling and endothelial dysfunction in aorta following HFD withdrawal. 8 weeks of HFD resulted in disturbed glucose homeostasis, endotheliual dysfunction, lipid remodeling and impaired sGC and insulin signaling in PVAT. HFD withdrawal led to early and late beneficial effects. Glucose tolerance was normalized as soon as 1 week of HFD withdrawal, which was accompanied by normalization of the decreased lipid unsaturation index in PVAT of AA, but not TA. In contrast, endothelial dysfunction in aorta was only partially reversed 1 week after HFD withdrawal; however, full restoration occurred 6 weeks after HFD withdrawal, which may be supported by substantial alterations in NO‐sGC‐cGMP and insulin signaling, and changes in adipokine expression, indicating their possible impact on the physiology of the vessel wall.

Our findings were summarized in Figure 6, underscoring the possible key role of sGC activity in maintaining PVAT homeostasis and supporting its potential as a pharmacological target for vascular dysfunction in obesity.

Moreover, metabolic disorders modulate endothelial dysfunction through metabolic memory, in which the adverse impacts of transiently abnormal metabolic conditions persist even after metabolic normalization [51]. Given this evidence, we cannot exclude that epigenetic changes in PVAT contribute to the delayed recovery of endothelial dysfunction to physiological levels after HFD withdrawal.

## 5 Limitations

This study provides time‐resolved and multimodal characterization of metabolic and vascular recovery following HFD withdrawal. By integrating in vivo MRI of acetylcholine‐induced vasodilation with intact PVAT, Raman spectroscopy, and RT‐qPCR, we were able to reveal a temporal dissociation between the early normalization of systemic insulin sensitivity and the delayed recovery of endothelial function in vivo, a finding not previously reported. This integrative and non‐destructive design represents a central strength of the study and underpins its translational relevance. Our work suggests that PVAT sGC may represent a novel mechanistic pathway and potential therapeutic target involved in vascular restoration after dietary fat reversal. Presented molecular data are limited to mRNA expression levels and do not identify cell‐specific changes that constrain mechanistic interpretation linking sGC and Et‐1 function in PVAT and endothelial function recovery in the aorta. Therefore, further studies are needed to explore our findings in more detail.

## 6 Conclusion

In conclusion, we demonstrated that HFD reversal quickly restored systemic insulin sensitivity but gradually improved endothelial function. Early restoration of insulin sensitivity, and PVAT lipid unsaturation was determined by *Scd1* and was temporarily dissociated from the reversal of endothelial dysfunction associated with persistent alterations in PVAT function that was ascribed to impaired guanylate cyclase signaling pathway. Therapeutic strategies aimed at preserving or enhancing NO–sGC–cGMP signaling, involving sGC‐mediated thermogenic pathways [34, 35], may provide therapeutic benefits in mitigating HFD‐induced PVAT dysfunction and enhancing recovery of endothelial function in metabolic diseases.

## Author Contributions

Conceptualization, K.C., M.S.F.‐A., and S.C.; sample preparation, K.C., I.C.‐C., E.S., and M.Z.P.; methodology, K.C., I.C.‐C., A.B. and M.W., formal analysis, K.C., I.C.‐C., A.B., and M.W.; investigation, K.C., I.C.‐C., A.B., E.S., M.W., Z.B., B.M., E.B.‐G. and P.P.‐G.; writing – original draft preparation, K.C.; writing – review and editing, K.C., M.S.F.‐A., and S.C.; visualization, K.C.; resources, K.C.; supervision, K.C. and S.C.; funding acquisition, K.C. All authors have read and agreed to the published version of the manuscript.

## Funding

This work was supported by the National Science Centre, Poland: OPUS27 (no. DEC‐2024/53/B/NZ5/03843 to K.C.) and Ministerio de Ciencia e Innovación (PID2022‐137116OB‐I00 to M.S.F.‐A.). K.C. was supported by the Federation of European Biochemical Societies (FEBS).

## Ethics Statement

All experimental procedures involving animals were approved by the Local Animal Ethics Commission (Krakow, Poland, identification code: 246/2023) and conducted according to the Guidelines for Animal Care and Treatment of the European Communities and the Guide for the Care and Use of Laboratory Animals published by the US National Institutes of Health (NIH Publication No. 85–23, revised 1996). All procedures were approved by the Local Ethical Committee on Animal Experiments.

## Conflicts of Interest

The authors declare no conflicts of interest.

## Supporting information


**Table S1:** Diet composition and nutrition facts.
**Table S2:** Primer sequences.
**Table S3:** Pair comparison of gene expression levels between the thoracic and abdominal PVAT and aorta.
**Figure S1:** Effects of 8 weeks of HFD and 6 weeks of HFD withdrawal on animal weight, epididymal white adipose tissue, liver, and plasma lipid profile. Alterations in mice body weight presented as body mass (A) and body weight change (B), eWAT mass (C), and liver mass (D, normalized to the weight of the mouse), total triacylglycerols (TG, E), total cholesterol (TCHOL, F), LDL fraction (G) and HDL fraction (H) in C57BL/6J mice fed an HFD for 8 weeks and after replacement of HFD for 1, 2, 4 and 6 weeks in comparison to C57BL/6J mice fed a standard AIN‐93G diet for 8 weeks. Data are represented as mean ± SD of 7–8 determinations per group. Statistics: A, C‐H: one‐way ANOVA with Tukey post hoc test; B: unpaired t‐test. *p < 0.05, **p < 0.01, ***p < 0.001. Elevated after HFD body weight and plasma lipids alterations were changed after HFD reversal to the AIN‐93G diet (normal diet) for 1 week up to 6 weeks. In all studied groups, body weight significantly increased in comparison to the control group (Fig. S1A and B). The mass of eWAT slightly increased after 8 weeks of HFD (by 30% in comparison to the normal diet) and increased significantly after 1 week of HFD reversal and then decreased with the duration of the diet reversal (Fig. S1C). Additionally, alteration in body weight was not associated with liver mass changes (Fig. S1D). HFD feeding caused a decrease in total triacylglycerol level that was restored in the first week of diet reversal to a similar level as the control group (Fig. S1E). Total plasma cholesterol, LDL, and HDL levels were increased by HFD were lowered to the control group level after 6 weeks of diet change (Fig. S1F‐H). Surprisingly, in our study, HFD led to a decrease in plasma triglycerides and an increase in HDL cholesterol without altering total cholesterol. Similar lipid profile alterations have been reported in certain mouse strains exposed to high‐fat diets i.e. *Podrini, C. et al. Mamm Genome 2013, 24, 240–251*, particularly depending on diet composition, duration of feeding, and strain‐specific metabolic adaptations. In mice, HFD does not consistently reproduce the classical human dyslipidemic pattern, and in some cases may result in reduced circulating triglycerides and elevated HDL due to altered lipoprotein metabolism and redistribution of triglycerides to peripheral tissues.
**Figure S2:** Average Raman spectra of TA and AA PVAT (A and B, respectively) of C57Bl/6J mice fed an HFD for 8 weeks and after replacement of HFD by AIN‐93G diet for 1, 2, 4 and 6 weeks in comparison to C mice fed a standard AIN‐93G diet for 8 weeks. Spectra were normalized in the 1800–1200 cm^−1^ spectral range. Prolonged 8 weeks of HFD feeding caused alterations in the Raman spectral profile of PVAT manifested by the decreased intensity of bands associated with lipid unsaturation, i.e. at 1657 and 1267 cm^−1^ assigned to the C=C stretching and = C‐H deformations in the hydrocarbon chain, and increased intensity of a band at 1441 cm^−1^ attributed to the CH bending vibrations of CH_2_/CH_3_ groups.

## Data Availability

The data that support the findings of this study are available online https://doi.org/10.57903/UJ/9L5ESE.
