# Supplementary material for: Temporal Dissociation of Impaired Glucose Tolerance, Adipose Lipid Remodeling and Endothelial Dysfunction in Aorta After HFD Withdrawal
Source: FASEB J. 2026 Apr 25;40:e71829. doi: 10.1096/fj.202600102RR (PMC13109807; doi:10.1096/fj.202600102RR)
Supplement: Supplementary file 1 — Table S1: Diet composition and nutrition facts. Table S2: Primer sequences. Table S3: Pair comparison of gene expression levels between the thoracic and abdominal PVAT and aorta. Figure S1: Effects of 8 weeks of HFD and 6 weeks of HFD withdrawal on animal weight, epididymal white adipose tissue, liver, and plasma lipid profile. Alterations in mice body weight presented as body mass (A) and body weight change (B), eWAT mass (C), and liver mass (D, normalized to the weight of the mouse), total triacylglycerols (TG, E), total cholesterol (TCHOL, F), LDL fraction (G) and HDL fraction (H) in C57BL/6J mice fed an HFD for 8 weeks and after replacement of HFD for 1, 2, 4 and 6 weeks in comparison to C57BL/6J mice fed a standard AIN‐93G diet for 8 weeks. Data are represented as mean ± SD of 7–8 determinations per group. Statistics: A, C‐H: one‐way ANOVA with Tukey post hoc test; B: unpaired t‐test. *p < 0.05, **p < 0.01, ***p < 0.001. Elevated after HFD body weight and plasma lipids alterations were changed after HFD reversal to the AIN‐93G diet (normal diet) for 1 week up to 6 weeks. In all studied groups, body weight significantly increased in comparison to the control group (Fig. S1A and B). The mass of eWAT slightly increased after 8 weeks of HFD (by 30% in comparison to the normal diet) and increased significantly after 1 week of HFD reversal and then decreased with the duration of the diet reversal (Fig. S1C). Additionally, alteration in body weight was not associated with liver mass changes (Fig. S1D). HFD feeding caused a decrease in total triacylglycerol level that was restored in the first week of diet reversal to a similar level as the control group (Fig. S1E). Total plasma cholesterol, LDL, and HDL levels were increased by HFD were lowered to the control group level after 6 weeks of diet change (Fig. S1F‐H). Surprisingly, in our study, HFD led to a decrease in plasma triglycerides and an increase in HDL cholesterol without altering total cholesterol. Similar lipi [file FSB2-40-e71829-s001.docx]

**Temporal dissociation of impaired glucose tolerance, adipose lipid remodeling and endothelial dysfunction after HFD withdrawal**

Krzysztof Czamara^1,*^, Izabela Czyzynska-Cichon^1^, Anna Bar^1^, Ewa Stanek^1^, Mateusz Wawro^2^, Marta Z. Pacia^1^, Zeinep. Berkimbayeva^1^, Brygida Marczyk^1^, Elvira Bragado-García^3^, Paloma Palma-Guzman^3^, Maria S. Fernandez-Alfonso^3^, Stefan Chlopicki^1,4^

^1^Jagiellonian University in Krakow, Jagiellonian Centre for Experimental Therapeutics (JCET), Krakow, Poland

^2^Jagiellonian University in Krakow, Department of Cell Biochemistry, Faculty of Biochemistry, Biophysics and Biotechnology, Kraków, Poland

^3^Universidad Complutense de Madrid, Instituto Pluridisciplinar and Faculty of Pharmacy, Madrid, Spain

^4^Jagiellonian University Medical College in Krakow, Department of Pharmacology, Krakow, Poland

Correspondence: krzysztof.czamara@uj.edu.pl

**Table S1. Diet composition and nutrition facts.**

| Ingredients (g/kg) | AIN-93G  (control diet) | | HFD  (60% kcal of fat + 1% of cholesterol) | |
| --- | --- | --- | --- | --- |
| Cornstarch | 484,17 | | 0,00 | |
| Casein (> 85% protein) | 191,29 | | 261,67 | |
| Maltodextrin | 119,56 | | 163,55 | |
| Sucrose | 65,81 | | 90,02 | |
| Soybean oil (without additives) | 23,91 | | 32,71 | |
| Fiber (α-cellulose) | 47,82 | | 65,42 | |
| Mineral mixture (AIN-93g-MX) | 33,48 | | 45,79 | |
| Vitamin blend (AIN-93-VX) | 9,56 | | 13,08 | |
| L-Cystine | 2,87 | | 3,93 | |
| Choline hydrogen tartrate | 2,39 | | 3,27 | |
| tert-butylhydroquinone | 0,01 | | 0,02 | |
| Lard | 19,13 | | 320,55 | |
| Cholesterol | 0,00 | | 10,00 | |
| **Nutrition facts** | gm% | kcal% | gm% | kcal% |
| Proteins | 19,42 | 20 | 26,56 | 20 |
| Carbonhydrates | 67,91 | 70 | 26,66 | 20 |
| Lipids | 4,3 | 10 | 35,33 | 60 |
| of which: saturated | 0,99 |  | 11,89 |  |
| monounsaturated | 1,72 |  | 17,93 |  |
| polyunsaturated | 1,41 |  | 3,95 |  |

**Table S2. Primer sequences.**

| Gene | Forward primer | Reverse primer |
| --- | --- | --- |
| *Polr2b* (HKG) | GGATTCTGGGAACGTCGGAG | CCGGAGTGATCTCATCGTCG |
| *Scd1* | TTGGGAGGCCTGTACGGGAT | GCCCAGAGCGCTGGTCATG |
| *Insr* | GAACGGCGGACCTATGGAGC | AGGGAGCTTCAGCCCTTTGA |
| *Irs1* | ACCAGCCCTTAGGCAGCAATG | GAGGAAGACGTGAGGTCCTGG |
| *Irs2* | TCTGCCAGCACCTATGCAAGC | TAGGAAAGTGAGCAGCAGCGT |
| *Pi3kr1* | AGTGTCCAAATACCAGCAGGATCA | TTGCTGTACCGCTCCTGGGT |
| *Edn1* | CAGGAAAAGAACTCAGGGCCCAA | TGCTCGGTTGTGCGTCAACT |
| *Gucy1b1* | GCGCGGACACCATGTACGG | GCAGCAGCCACCAGGTCATA |
| *Nos3* | GCGTTTGATCCCCGGGTCCT | TCCATGAGCGCTGCTGCAAA |
| *Nox2* | TTCTCAGGGGTTCCAGTGCG | TGCAATTGTGTGGATGGCGGT |
| *Nox4* | GGGCGATTGTGTTTAAGCAGAGC | GCGGCTACATGCACACCTGA |
| *Lep* | AGCTGCAAGGTGCAAGAAGAA | GGAATGAAGTCCAAGCCAGTGAC |
| *Adipoq* | GATCTGACGACACCAAAAGGGCT | AAGTTCCCTTGGGTGGAGGGA |
| *Nampt* | GCCACCGACTCGTACAAGGTTA | ACACTTCTTTGGCCTCCTGGAT |
| *Ucp1* | GCTTGTCAACACTTTGGAAAGGGAC | GTGGTGCAAAACCCGGCAAC |
| *Pparg2* | AGAGCATGGTGCCTTCGCTG | CCGAAGTTGGTGGGCCAGAA |
| *Tnf* | GCTTCCAGAACTCCAGGCGG | TACGACGTGGGCTACAGGCT |

**Table S3. Pair comparison of gene expression levels between the thoracic and abdominal PVAT and aorta.**

| Gene | TA vs AA  Ctrl | TA vs AA  HFD | TA vs. AA  HFD withdrawal  1 week | TA vs AA  HFD withdrawal  6 week | |
| --- | --- | --- | --- | --- | --- |
| **PVAT** | | | | |  |
| *Scd1* | (-0,323 ± 0,142)  * | (-0,015 ± 0,017)  ns | (-0,340 ± 0,088)  ** | (-0,111 ± 0,095)  ns | |
| *Insr* | (0,342 ± 0,151)  * | (0,566 ± 0,142)  ** | (0,291 ± 0,165)  ns | (0,148 ± 0,116)  ns | |
| *Irs1* | (0,128 ± 0,165)  ns | (0,205 ± 0,090)  * | (0,100 ± 0,184)  ns | (-0,382 ± 0,137)  * | |
| *Irs2* | (-0,199 ± 0,190)  ns | (-0,713 ± 0,286)  * | (-0,593 ± 0,223)  * | (-0,693 ± 0,176)  ** | |
| *Pi3kr1* | (-0,019 ± 0,141)  ns | (0,111 ± 0,086)  ns | (0,112 ± 0,192)  ns | (0,098 ± 0,136)  ns | |
| *Edn1* | (-0,262 ± 0,216)  ns | (0,010 ± 0,056)  ns | (-0,010 ± 0,036)  ns | (-0,201 ± 0,148)  ns | |
| *Gucy1b1* | (0,397 ± 0,219)  ns | (0,183 ± 0,045)  ** | (0,114 ± 0,065)  ns | (0,512 ± 0,307)  ns | |
| *Ucp1* | (-0,392 ± 0,106)  ** | (-0,381 ± 0,084)  ** | (-0,273 ± 0,147)  ns | (-0,155 ± 0,082)  ns | |
| *Pparg2* | (-0,229 ± 0,122)  * | (-0,248 ± 0,080)  * | (-0,204 ± 0,113)  ns | (-0,171 ± 0,079)  ns | |
| *Tnf* | (0,215 ± 0,128)  ns | (0,011 ± 0,322)  ns | (0,157 ± 0,086)  ns | (0,209 ± 0,082)  ns | |
| *Lep* | (2,838 ± 0,771)  ** | (2,848 ± 0,995)  * | (4,393 ± 0,914)  ** | (0,116 ± 0,615)  ns | |
| *Adipoq* | (0,891 ± 0,153)  *** | (0,594 ± 0,166)  ** | (0,669 ± 0,103)  *** | (0,830 ± 0,089)  **** | |
| *Nampt* | (0,395 ± 0,218)  ns | (0,348 ± 0,148)  * | (0,863 ± 0,303)  * | (0,345 ± 0,181)  ns | |
| **Aorta** | | | | |  |
| *Insr* | (0,270 ± 0,090)  * | (0,091 ± 0,091)  ns | (-0,106 ± 0,052)  ns | (0,106 ± 0,104)  ns | |
| *Irs1* | (0,687 ± 0,117)  *** | (0,883 ± 0,145)  *** | (0,568 ± 0,120)  *** | (0,829 ± 0,227)  ** | |
| *Irs2* | (0,052 ± 0,049)  ns | (0,043 ± 0,061)  ns | (0,039 ± 0,077)  ns | (0,116 ± 0,039)  ns | |
| *Pi3kr1* | (0,958 ± 0,169)  *** | (0,504 ± 0,204)  * | (0,812 ± 0,118)  **** | (0,939 ± 0,255)  ** | |
| *Nos3* | (0,831 ± 0,262)  * | (1,008 ± 0,169)  *** | (0,407 ± 0,185)  ns | (0,708 ± 0,131)  *** | |
| *Gucy1b1* | (-0,032 ± 0,023)  ns | (0,006 ± 0,067)  ns | (-0,165 ± 0,049)  ** | (-0,077 ± 0,048)  ns | |
| *Nox2* | (0,635 ± 0,080)  **** | (0,940 ± 0,211)  ** | (0,778 ± 0,197)  ** | (0,522 ± 0,301)  ns | |
| *Nox4* | (0,306 ± 0,099)  * | (0,257 ± 0,097)  * | (0,155 ± 0,067)  * | (0,397 ± 0,145)  * | |
| *Edn1* | (0,261 ± 0,181)  ns | (0,440 ± 0,214)  ns | (0,809 ± 0,226)  ** | (0,472 ± 0,193)  * | |

Difference between means (AA-TA) ± SEM. *P<0.05, **P<0.01, ***P<0.001, ****P<0.0001


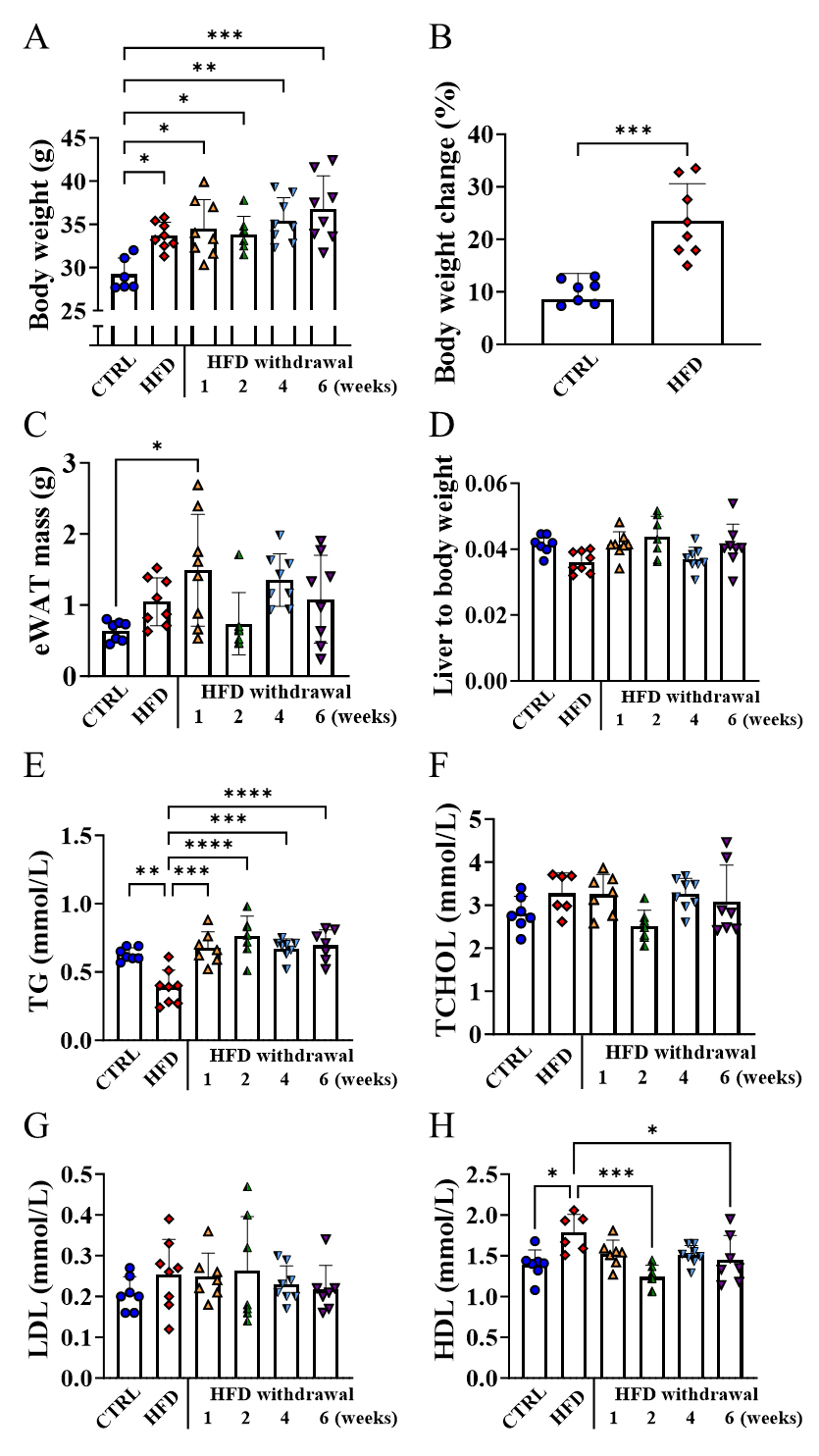


**Fig. S1. Effects of 8 weeks of HFD and 6 weeks of HFD withdrawal on animal weight, epididymal white adipose tissue, liver, and plasma lipid profile.** Alterations in mice body weight presented as body mass (A) and body weight change (B), eWAT mass (C), and liver mass (D, normalized to the weight of the mouse), total triacylglycerols (TG, E), total cholesterol (TCHOL, F), LDL fraction (G) and HDL fraction (H) in C57BL/6 mice fed an HFD for 8 weeks and after replacement of HFD for 1, 2, 4 and 6 weeks in comparison to C57BL/6 mice fed a standard AIN-93G diet for 8 weeks. Data are represented as mean ± SD of 7-8 determinations per group. Statistics: A, C-H: one-way ANOVA with Tukey post hoc test; B: unpaired t-test. *p<0.05, **p<0.01, ***p<0.001.

Elevated after HFD body weight and plasma lipids alterations were changed after HFD reversal to the AIN-93G diet (normal diet) for 1 week up to 6 weeks. In all studied groups, body weight significantly increased in comparison to the control group (Fig. S1A and B). The mass of eWAT slightly increased after 8 weeks of HFD (by 30% in comparison to the normal diet) and increased significantly after 1 week of HFD reversal and then decreased with the duration of the diet reversal (Fig. S1C). Additionally, alteration in body weight was not associated with liver mass changes (Fig. S1D). HFD feeding caused a decrease in total triacylglycerol level that was restored in the first week of diet reversal to a similar level as the control group (Fig. S1E). Total plasma cholesterol, LDL, and HDL levels were increased by HFD were lowered to the control group level after 6 weeks of diet change (Fig. S1F-H). Surprisingly, in our study, HFD led to a decrease in plasma triglycerides and an increase in HDL cholesterol without altering total cholesterol. Similar lipid profile alterations have been reported in certain mouse strains exposed to high-fat diets i.e*. Podrini, C. et al. Mamm Genome 2013, 24, 240–251*, particularly depending on diet composition, duration of feeding, and strain-specific metabolic adaptations. In mice, HFD does not consistently reproduce the classical human dyslipidemic pattern, and in some cases may result in reduced circulating triglycerides and elevated HDL due to altered lipoprotein metabolism and redistribution of triglycerides to peripheral tissues.

**Fig. S2.** Average Raman spectra of TA and AA PVAT (A and B, respectively) of C57Bl/6 mice fed an HFD for 8 weeks and after replacement of HFD by AIN-93G diet for 1, 2, 4 and 6 weeks in comparison to C57BL/6 mice fed a standard AIN-93G diet for 8 weeks. Spectra were normalized in the 1800-1200 cm^-1^ spectral range.

Prolonged 8 weeks of HFD feeding caused alterations in the Raman spectral profile of PVAT manifested by the decreased intensity of bands associated with lipid unsaturation, i.e. at 1657 and 1267 cm^-1^ assigned to the C=C stretching and =C-H deformations in the hydrocarbon chain, and increased intensity of a band at 1441 cm^-1^ attributed to the CH bending vibrations of CH_2_/CH_3_ groups.
